# Water buffalo as sentinel animals for schistosomiasis surveillance

**DOI:** 10.2471/BLT.14.143065

**Published:** 2015-05-28

**Authors:** Jose Ma M Angeles, Lydia R Leonardo, Yasuyuki Goto, Masashi Kirinoki, Elena A Villacorte, Hassan Hakimi, Kharleezelle J Moendeg, Seungyeon Lee, Pilarita T Rivera, Noboru Inoue, Yuichi Chigusa, Shin-ichiro Kawazu

**Affiliations:** a National Research Center for Protozoan Diseases, Obihiro University of Agriculture and Veterinary Medicine, Nishi 2–13, Inada-cho Obihiro, Hokkaido, Japan 080–8555.; b Department of Parasitology, University of the Philippines, Manila, Philippines.; c Laboratory of Molecular Immunology, University of Tokyo, Tokyo, Japan.; d Laboratory of Tropical Medicine and Parasitology, Dokkyo Medical University, Tochigi, Japan.

## Introduction

About 75% of human pathogens are zoonotic, meaning that they are communicated from animals to humans.^1^ Schistosomiasis is a zoonotic disease with a complex transmission cycle involving aquatic snails and least 40 species of mammals, which serve as reservoir hosts. Worldwide, more than 700 million people are at risk of schistosomiasis and over 240 million people are infected with the parasite.^2^ Schistosomiasis has been eliminated in Japan and the coastal plains of China by a combination of medical treatment, health education, improved water quality and sanitation and snail control through environmental modification, molluscicide and new farming methods. The World Health Organization (WHO) targets elimination of schistosomiasis in the Eastern Mediterranean, the Caribbean, Indonesia and the Mekong river basin by 2015, and in the Western Pacific, the Americas and selected African countries by 2020.^3^ Here we argue that elimination guidelines for schistosomiasis should include surveillance of the animal reservoir.

## Transmission patterns

Mapping of the transmission patterns in humans and animals can lead to a better understanding of transmission of schistosomiasis between different host species. Parts of China, Indonesia and the Philippines are endemic for schistosomiasis caused by *Schistosoma japonicum*. Cattle, water buffalo, goats, dogs, pigs and rats are potentially important reservoir hosts for this parasite because of their contact with humans. Other animals including cats and horses are not significant contributors because of their limited contact with contaminated water.^4^

Grazing ruminants including goats, cattle and water buffalo are exposed to the parasite in transmission sites such as rice paddies. However, goats do not contribute much to transmission as they are uncommon in areas where schistosomiasis is endemic. Cattle and water buffalo are the major animal reservoirs contributing to human transmission of *S. japonicum*.^4^^–^^7^ In China, bovine defecation is thought to be the main cause of environmental contamination.^6^ Molecular studies have also suggested that transmission is through bovines rather than other domesticated animals.^7^ Cattle are less capable of recovering from infection than water buffalo.^8^ Despite the fact that buffalo are capable of clearing the parasite, they are a major reservoir host because they are repeatedly exposed to the parasite when pulling ploughs in rice paddies.^9^

In rice-growing regions, water buffalo are a good choice of sentinel animal. In Anhui and Sichuan provinces of China, re-emergence of schistosomiasis in humans was attributed to the high prevalence of schistosome infection among cows and water buffalo.^10^^,^^11^ These human infections could be avoided by setting up a sensitive animal surveillance system to detect infection in animals and, by treating them promptly, preventing further contamination of the environment.

## Diagnostic tests

Microscopic examination of stool samples is a standard test for several parasitic diseases, including schistosomiasis. However, microscopy has low sensitivity in diagnosing schistosomiasis among water buffalo due to the large volume of bovine excreta. Therefore, a more sensitive and specific diagnostic test is needed to identify schistosomiasis-free areas and prevent re-emergence of the disease. In previous studies, polymerase chain reaction (PCR)^12^ and recombinant antigen-based enzyme-linked immunosorbent assay (ELISA)^13^ have been found to be useful in identifying *S. japonicum* in stool samples. Surveillance can also be carried out in abattoirs where cattle and buffalo are slaughtered. Documentation of the original source of livestock then allows schistosome-infested sites to be located.

## Medical and veterinary approaches

The complexity of zoonotic schistosomiasis transmission has been a major hindrance to elimination of the disease. Definitive, intermediate and reservoir host factors as well as social and environmental influences should all be considered in the control of this disease. An integrated approach with the cooperation of human and animal health agencies will improve the chances of success.^14^

Current control strategies for schistosomiasis include mass drug administration and health education of human hosts. Japan eliminated schistosomiasis before praziquantel was available through snail control measures including molluscicides, construction of cement ditches and land reclamation. However due to the toxicity of molluscicides and the variety of habitats occupied by intermediate host snails, large-scale snail control is not safe or practical. Disease control in the animal reservoir has also been shown to be cost–effective in other zoonotic diseases, including brucellosis^15^ and rabies.^16^

In working towards the elimination of schistosomiasis, selective treatment or isolation of infected animals may be the most practical approach. Providing toilets and health education to reduce open defecation by humans would also reduce the risk of infection for both humans and animals. Veterinarians and public health professionals need to work together in the battle against zoonotic schistosomiasis.
